# Geographical Distribution and Genetic Diversity of the Banana Fusarium Wilt Fungus in Laos and Vietnam

**DOI:** 10.3390/jof8010046

**Published:** 2022-01-02

**Authors:** Khonesavanh Chittarath, Chung Huy Nguyen, Wendy C. Bailey, Si-Jun Zheng, Diane Mostert, Altus Viljoen, Anthony Fredrick Tazuba, Walter Ocimati, Elizabeth Kearsley, Trần Yến Chi, Nguyen Thi Tho, Nguyen Tien Hung, Miguel Dita, Trushar Shah, Margaret Karanja, George Mahuku, Guy Blomme

**Affiliations:** 1 Plant Protection Center, Department of Agriculture, Ministry of Agriculture and Forestry, Vientiane 01001, Laos; chittarhat_2005@yahoo.com; 2 Plant Protection Research Institute, Duc Thang Commune, Bac Tu Liem District, Hanoi 119000, Vietnam; hchungvasi@yahoo.com (C.H.N.); ntho57.ppri@gmail.com (N.T.T.); hungnguyen1218@gmail.com (N.T.H.); 3 Department of Plant Pathology, Stellenbosch University, Stellenbosch 7602, South Africa; wbailey@sun.ac.za (W.C.B.); diane@sun.ac.za (D.M.); altus@sun.ac.za (A.V.); 4 Yunnan Key Laboratory of Green Prevention and Control of Agricultural Transboundary Pests, Agricultural Environment and Resources Institute, Yunnan Academy of Agricultural Sciences, Kunming 650205, China; s.zheng@cgiar.org; 5 The Alliance of Bioversity and CIAT, Kunming 650205, China; 6 The Alliance of Bioversity and CIAT, Kampala P.O. Box 24384, Uganda; tazubatony@gmail.com (A.F.T.); w.ocimati@cgiar.org (W.O.); 7 BlueGreen Labs, 9120 Melsele, Belgium; kearsleyelizabeth@gmail.com; 8 Plant Protection Department, 149 Ho Dac Di, Dong Da, Hanoi 115000, Vietnam; yenchitran84@gmail.com; 9 The Alliance of Bioversity and CIAT, Cali-Palmira CP 763537, Colombia; m.dita@cgiar.org; 10 IITA, Nairobi P.O. Box 30709-00100, Kenya; tm.shah@cgiar.org (T.S.); m.karanja@cgiar.org (M.K.); 11 IITA, Kampala P.O. Box 7878, Uganda; g.mahuku@cgiar.org; 12 The Alliance of Bioversity and CIAT, Addis Ababa P.O. Box 5689, Ethiopia

**Keywords:** Cavendish, field survey, Fusarium TR4, Greater Mekong Subregion, ‘Pisang Awak’

## Abstract

Fusarium wilt, caused by the fungus *Fusarium oxysporum* f. sp. *cubense* (Foc), poses a major threat to global banana production. The tropical race 4 (TR4) variant of Foc is a highly virulent form with a large host range, and severely affects Cavendish bananas. Foc TR4 was recently observed within the Greater Mekong Subregion, after Chinese private companies expanded Cavendish production to the region. In this study, extensive surveys conducted across Laos and Vietnam show that Foc TR4 is still mainly constricted to the northern regions of these countries and is limited to Cavendish cultivation settings. In Laos, Foc TR4 is associated with large-scale Cavendish plantations owned by or involved with Chinese companies through which infected planting material could have been imported. In Vietnam, mostly small-holder Cavendish farmers and backyard gardens were affected by Foc TR4. In Vietnam, no direct link is found with Chinese growers, and it is expected the pathogen mainly spreads through local and regional movement of infected planting materials. Foc TR4 was not recorded on banana cultivars other than Cavendish. The extensively cultivated ‘Pisang Awak’ cultivar was solely infected by VCGs belonging to Foc race 1 and 2, with a high occurrence of VCG 0123 across Laos, and of VCG 0124/5 in Vietnam. Substantial diversity of Foc VCGs was recorded (VCGs 0123, 0124/5, 01218 and 01221) from northern to southern regions in both countries, suggesting that Fusarium wilt is well established in the region. Interviews with farmers indicated that the local knowledge of Fusarium wilt epidemiology and options for disease management was limited. Clear communication efforts on disease epidemiology and management with emphasis on biosecurity practices need to be improved in order to prevent further spread of Foc TR4 to mixed variety smallholder settings.

## 1. Introduction

Fusarium wilt is a major disease of banana (*Musa* spp.) found in most banana-growing countries [1,2,3,4]. The disease is caused by a soil-borne ascomycete fungus, *Fusarium oxysporum* f. sp. *cubense* (Foc), which evolved with its host in Southeast Asia. Foc infects the roots of banana, then blocks the vascular system of the plant, thereby causing wilting and eventual death [5,6]. The fungus reproduces strictly asexually and can survive as chlamydospores in infested banana fields for up to 30 years. It also survives as an asymptomatic endophyte in alternative host plants such as weeds [7,8,9,10,11]. Foc is spread locally and regionally with infected planting material and infested soil and water [11].

Foc is a highly diverse pathogen that comprises different evolutionary lineages. The fungus is classified into three physiological races based on its pathogenicity to differential host cultivars, with Foc race 1 (R1) affecting ‘Gros Michel’ (AAA), ‘Lady Finger’ (AAB) and ‘Pisang Awak’ (ABB) bananas; Foc race 2 (R2) affecting ‘Bluggoe’ (ABB) and closely related ABB clones; and Foc race 4 (R4) affecting the ‘Cavendish’ banana subgroup (AAA) as well as most cultivars susceptible to Foc R1 and R2 [3,6,12,13]. However, the race structure is often confusing and inaccurate in delineating Foc strains. To improve the identification of Foc strains, heterokaryon formation between Foc isolates is used to further divide the fungus into 24 vegetative compatibility groups (VCGs) [12,14,15].

Foc R4 has been divided into ‘subtropical’ race 4 (SR4) and ‘tropical’ race 4 (TR4) strains, based on their ability to cause disease to Cavendish bananas under different environmental conditions [16,17]. Foc SR4 strain infects Cavendish bananas in the subtropics only, whilst Foc TR4 affects Cavendish in both the tropics and subtropics [12,18]. Phylogenetically, Foc SR4 strains consist of VCGs 0120/15, 0122, 0126, 0129/11, 01210 and 01219, whereas Foc TR4 includes the VCG complex 01213/16 and 0121 [4,19,20].

Foc TR4 is considered to be the most damaging form of Foc due to its wide host range and severe effect to Cavendish bananas, which constitute about 50% of all bananas produced globally [21]. The fungus was initially restricted to Southeast Asia and Australia until approximately 2012 [9,22,23,24,25], whereafter it has been detected in the Middle East [10,26,27], Mozambique and Mayotte in Africa [28,29], and Colombia and Peru in Latin America [30,31]. Foc TR4 also spread to new countries in Asia, most likely from China [23]. Since 2018 the fungus was reported in Laos [23,32], Vietnam [23,33], Myanmar and Cambodia [23] and Thailand [34]. These detections occurred after Chinese private companies expanded Cavendish production in the Greater Mekong Subregion (Laos, Vietnam, Myanmar and Cambodia) following a decline in banana production in southern China due to Fusarium wilt [23]. The private companies frequently transport planting materials and relocate farm equipment from infested areas in China into the Greater Mekong Subregion countries, thereby increasing the risks of disseminating Foc TR4 [10].

In this study, the distribution and diversity of Foc in banana fields in Laos and Vietnam were investigated through field surveys. Laos and Vietnam are of specific interest for Fusarium wilt surveys, since banana production in both countries has increased significantly from 1.6 to 2.2 Mt in northern and southern Vietnam, and from 0.18 to 1.06 Mt in Laos, over the past decade [21]. During this period, banana cultivation has also shifted from local dessert bananas towards the production of Cavendish bananas, which currently represents 80% of bananas in Vietnam (Personal communication, 2021, Deputy director of Fruit and Vegetable Research Institute, Vietnam) and 85% of all bananas grown in Laos [35]. The objectives of the surveys were to (a) collect, conserve, and characterize Foc isolates from Laos and Vietnam, (b) assess the genetic diversity and distribution of *Musa* varieties affected by banana Fusarium wilt (TR4 and other races), and (c) determine the status and extent of Foc TR4 infestation in Laos and Vietnam for future mitigation and disease prevention.

## 2. Materials and Methods

### 2.1. Study Area and Field Surveys

**Figure 1:**
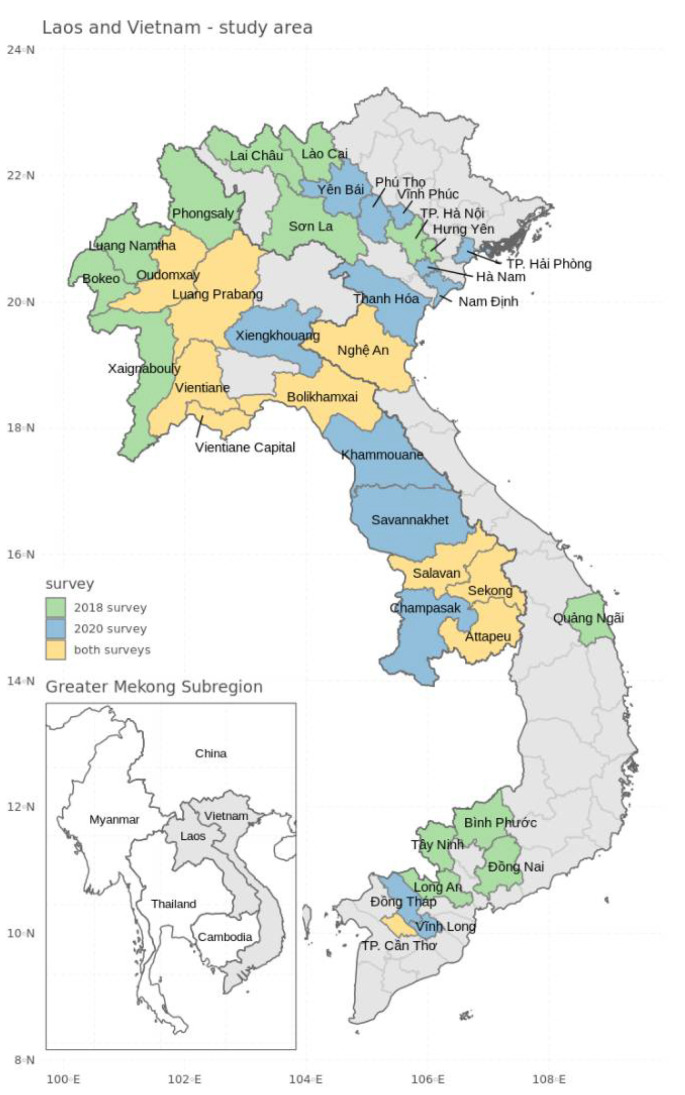
Laos and Vietnam survey area. Provinces that were surveyed in 2018 are indicated in green, and the provinces that were surveyed in 2020 are indicated in blue. The provinces that were surveyed during both years are indicated in yellow.

Two field surveys were organized across Laos and Vietnam to determine the presence of banana Fusarium wilt. The first survey was performed in 12 provinces in Vietnam between September 2018 and February 2019, and in 12 provinces in Laos between September 2018 and October 2019 (Figure 1). These surveys solely focused on Cavendish banana fields and cultivation zones. The Cavendish cultivar was selected because of its extreme susceptibility to Foc TR4, and because Foc TR4 was anticipated to have been introduced with infected planting materials and imported machinery by Chinese Cavendish banana-producing companies. A second survey was performed between September and November 2020. This survey also included all banana cultivars and small-holder farms that could have been infected through local trade and contact with Foc TR4-affected Cavendish farms. Eight provinces in Laos and two provinces in Vietnam that were part of the 2018 survey were again visited, as well as an additional four and nine provinces in Laos and Vietnam, respectively (Figure 1).

Field surveys and sample collection activities were carried out on large banana plantations, smallholder farms and backyard gardens where symptoms reminiscent of Fusarium wilt were observed. In the 2018 survey, one to five districts per province were selected for sampling, with a total of 27 districts, 46 villages and 155 fields in Laos, and 40 districts, 71 villages and 147 fields in Vietnam (Table 1). One to five symptomatic banana plants were sampled in each field, depending on the number of symptomatic plants. The 2020 survey covered 27 districts, 96 villages and 120 fields in Laos, and 30 districts, 62 villages and 85 fields in Vietnam (Table 2). During this survey, one to three symptomatic banana mats were sampled per field. During both surveys, sampling consisted of the collection of banana pseudostem tissues from banana plants that showed characteristic Foc symptoms of leaf yellowing as described by Viljoen et al. [36]. Field sample collection data were collated into spread sheets using the Open Data Kit tool [37]. These data were subsequently curated and imported into the ONA platform (https://company.ona.io/, accessed on 3 September 2021). This platform has been used to integrate and share the data collected by the different teams onto a common platform. The sample collection data for the 2018 and 2020 surveys have been combined and are available for download and visualization at the following link https://ona.io/seedtracker/149457/618308#/table, accessed on 3 September 2021. The field level detailed survey data for the 2020 survey for both Laos and Vietnam, respectively, are available at the following links https://ona.io/seedtracker/149457/602284#/table, accessed on 3 September 2021 and https://ona.io/seedtracker/149457/585678#/table, accessed on 3 September 2021. Post-processing was performed using R version 3.6.3 [38].

During the 2020 survey, farmers were also interviewed about the presence of Fusarium wilt in their banana fields and backyard gardens, the constraints this disease and other biotic factors have caused to their banana production and which disease mitigation and control measures were applied. Additional questions were asked about their overall knowledge on fusarium wilt, including how it spreads, how it affects the banana plant and overall management.

### 2.2. 2018 Survey: Sample Processing and Detection of Foc

Fungal isolation: The isolation of Foc TR4 from discolored pseudostem samples was conducted at the Plant Quarantine Diagnostic Centre (PQDC) in Hanoi, Vietnam, and at the Department of Agriculture and Forestry in Vientiane Capital, Laos. In the laboratory, Foc isolation was conducted as described by Dita et al. [39], García-Bastidas et al. [24] and Viljoen et al. [36]. Pseudostem samples were first surface sterilized, and primary isolations were performed by plating out 5 mm strands of infected vascular tissue on potato dextrose agar (PDA). After 3 days, fungal colonies suggestive of *Fusarium* spp. were sub-cultured onto PDA plates and incubated at 25 °C. After 7 to 10 days, pure cultures were obtained from cultures with Foc characteristic morphological features by further sub-culturing using hyphae tipping onto PDA plates and incubating as described above. The purified cultures were then stored on PDA at −4 °C for short-term storage and in 30% glycerol at −80 °C for long term storage.

Genomic DNA extraction: Fungal mycelium of each isolate was harvested by carefully scraping it off the PDA agar with sterile scalpel blades, and depositing it into separate, labeled, sterile 1.5 mL micro-centrifuge tubes. Total fungal genomic DNA of the Vietnam and Laos samples were extracted and purified using the Fair Biotech Genomic DNA Isolation and Purification kit (Fair Biotech, Taoyuan, Taiwan) and the PureDireX kit (Bio-Helix, Keelung, Taiwan), respectively. The eluted gDNA samples were then quantified using a NanoDrop^TM^ spectrophotometer (NanoDrop, Wilmington, DE, USA), diluted to 10 ng/µL, and stored at −20 °C for subsequent use.

Molecular characterization by PCR analysis: Molecular identification of Foc TR4 was conducted by a duplex PCR amplification, using a 648-bp primer specific to the detection of the translation elongation factor 1-α (TEF1-α) as an internal control gene [40] and a 463-bp Foc TR4-specific primer [39]. The PCR amplicons were electrophoresed on a 1.2% (*w*/*v*) agarose gel and visualized using an UV illuminator. Genomic DNA of a reference strain of VCG 01213/16 [33] was used as a positive control, whereas for the no template control the genomic DNA was substituted with nuclease-free water. Foc isolates for the 2018 survey were not characterized at VCG level.

### 2.3. 2020 Survey: Sample Processing and Detection of Foc

Fungal isolation: Four vascular strands of 5 mm each were cut from each sample and plated onto PDA supplemented with streptomycin (4 mg/L) in 90-mm-diameter Petri dishes. The Petri dishes were incubated for 7 days at 25 °C, after which the cultures were purified and single-spored. Only samples that resembled *Fusarium* spp. in culture were stored on carnation leaf agar (CLA) slants at 4 °C and in 30% glycerol at −80 °C in the culture collection of the Department of Plant Pathology at Stellenbosch University, South Africa.

DNA extraction: The *Fusarium* spp. isolates were grown on PDA at 25 °C for 5 days, before mycelium was harvested by scraping it off the surface with a sterile scalpel and depositing it into 2 mL Eppendorf tubes. The mycelia were then lyophilized in a VirTis BenchTop Pro freeze dryer (Warminster, PA, USA), and DNA extractions performed as described by González-Mendoza et al. [41]. The quality and quantity of DNA was determined with a NanoDrop Nd-1000 Spectrophotometer (Thermo Scientific, Waltham, MA, USA), and stored at −20 °C until use.

Molecular identification with PCR: All *Fusarium* sp. isolates were systematically identified using molecular markers (Supplementary Figure S1). The DNA was first PCR amplified with Foc TR4-specific primers described by Dita et al. [39] and Matthews et al. [42]. If negative, the DNA was PCR amplified with Foc Clade A-specific primers (Mostert, unpublished data) to determine if the isolates belong to VCGs 0120/15, 0121, 0122, 0126, 0129/11, 01210 and 01219 [43]. If again negative, primers specific to Foc Lineage VI was used for PCR amplification, as described by Ndayihanzamaso et al. [44] and Matthews et al. [42]. Isolates that were negative for all three PCR amplifications were subjected to PCR assays specific to Foc Lineage VII, VCG 01218 and VCG 01221 (Mostert, unpublished data). *Fusarium* isolates that were not amplified by any of the Foc primer sets were considered either new Foc VCGs, non-pathogenic *F. oxysporum*, or unknown *Fusarium* species.

Vegetative compatibility group testing: VCG analysis [45] was performed to confirm the identities of Foc isolates that were previously identified using molecular markers. The Foc isolates were first grown on PDA for 7 days at 25 °C, and the hyphae of actively growing cultures transferred onto minimal media (MM) supplemented with 1–3% KClO_3_. These cultures were then incubated for 7–21 days at 25 °C to generate ClO_3_-resistant mutants. Sectors of sparse mycelial growth colonies were considered nitrate non-utilizing (*nit*)-mutants, and were phenotyped as *nit*-1, *nit*-3 or Nit-M mutants [45]. The VCG identities of Foc isolates from Laos and Vietnam were then determined by pairing *nit*-1 and *nit*-3 mutants with Nit-M testers of known VCGs. Isolates earlier identified as Foc TR4 using molecular markers were paired with VCGs 01213, 01216 and 01213/16, and those that amplified with Lineage VI-specific primers were tested against VCGs 0124, 0125, 0128, 01212, 01220 and 01222. The isolates that tested positive for Lineage VII were paired with VCGs 0123, 01217, 01223 and 01224. If the isolates produced amplification products specific to VCG 01218 and VCG 01221, they were paired with these testers. The unknown isolates were designated a specific VCG if their *nit*-mutants formed heterokaryons with Nit-M mutants of VCG tester strains [45]. When *nit*-1 and *nit*-3 mutants of a particular isolate failed to pair with its own Nit-M mutants, the isolate was considered heterokaryon self-incompatible. All the pairings were repeated at least once.

## 3. Results

**Figure 2:**
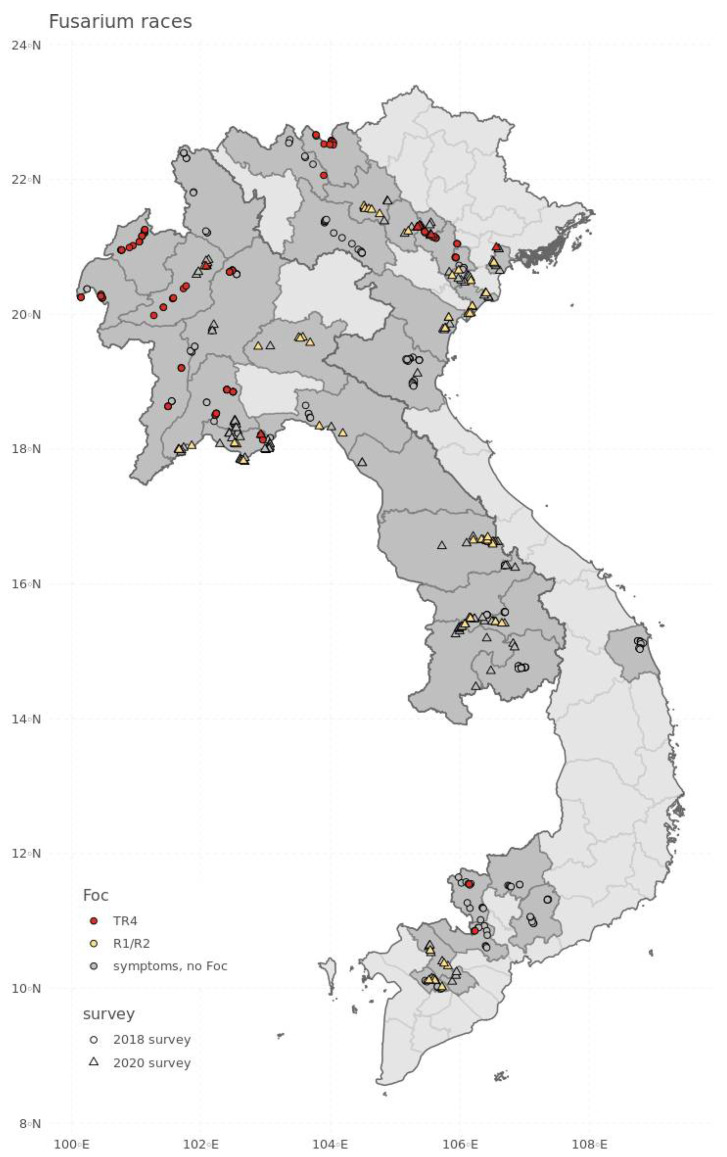
Observations of *Fusarium oxysporum* f. sp. *cubense* (Foc) TR4 and Foc R1/R2. Fields surveyed in 2018 are indicated as circles, whereas fields surveyed in 2020 are marked as triangles. Grey points show fields with banana plants showing the traditional symptoms of wilt, but were not identified as fusarium; red points denote sites where Foc TR4 was positively identified; yellow points indicate sites where Foc R1/R2 was positively identified.

Fusarium wilt was widely found in the northern regions of Laos and Vietnam, the central and southern regions of Laos and the southern regions of Vietnam (Figure 2). Of a total of 507 fields in which symptomatic plants were inspected and sampled during 2018 and 2020, positive identifications of Fusarium wilt have been made in 148 fields, indicating that these fields have at least one banana plant infected with Foc. Accordingly, in 29.2% of fields with symptomatic plants, the symptoms of wilting were confirmed, using a Foc TR4-specific primer [39] (Supplementary Figure S2) and VCGs (Supplementary Figure S3) to be caused by Foc TR4. Most of the Foc TR4-infected Cavendish bananas were recorded in the northern parts of Laos and Vietnam (Figure 2, Table 1).

In Laos, a substantial number of Cavendish fields were infected with TR4 in the northwestern provinces of Bokeo (71.4% of fields with symptomatic plants), Luang Namtha (100%), Oudomxay (60%), Xaignabouly (70%) and Luang Prabang (30%) (Table 1) and the central provinces of Vientiane (59.1%) and Vientiane Capital (20%). None of the Cavendish fields in the southern provinces of Laos were infected with Foc TR4. In Vietnam, Foc TR4 infected Cavendish fields were observed in the northwestern provinces of Lao Cai (80%) and Lai Chau (14.3%) (Table 1), and in the provinces Hanoi (50%) and Hung Yen in the Red River Delta (36.4%). Two additional Foc TR4-infested Cavendish fields were identified in the 2020 survey in the Red River Delta region and three in the northeastern province of Phu Tho (Table 2). In the southern parts of Vietnam, two Foc TR4-infested Cavendish fields were observed in the provinces of Tay Ninh and Long An, respectively (Figure 2, Table 1).

In 2020, ‘Pisang Awak’ (ABB) was the banana variety most affected by Fusarium wilt. Symptomatic ‘Pisang Awak’ plants were collected from 76 fields, four of which also had symptomatic Cavendish plants. Symptomatic Cavendish plants were collected in an additional four fields, symptomatic ‘Pisang Mas’ (AA, Subgroup Sucrier) plants from two fields and symptomatic triploid BBB cooking banana from three fields in Vietnam. In Laos, symptomatic ‘Pisang Awak’ plants were found in 118 fields, and symptomatic Cavendish plants in only two fields.

Foc R1/R2 was recorded in all regions of Laos and Vietnam (Figure 2). In Laos, Foc R1/R2 was found in the northwestern provinces of Oudomxay (9.1%) and Xiangkhouang (83.3%) (Table 2), the central provinces of Vientiane Capital (10.7%), Vientiane (13.3%), Bolikhamxai (66.7%) and Savannakhet (47.4%) and the south-eastern provinces of Champasak (10%), Salavan (10%) and Sekong (20%). In Vietnam, Foc R1/R2 found in the northwestern province of Yen Bai (50%), the northeastern province of Phu Tho (45.5%), the provinces Ha Nam (30%), Hai Phong (20%), Nam Dinh (45.5%) and Vin Phuc in the Red River Delta (14.3%), the central province Thanh Hoa (42.9%) and the southern provinces Can Tho (71.4%) and Dong Thap (37.5%) in the Mekong River Delta.

**Figure 3:**
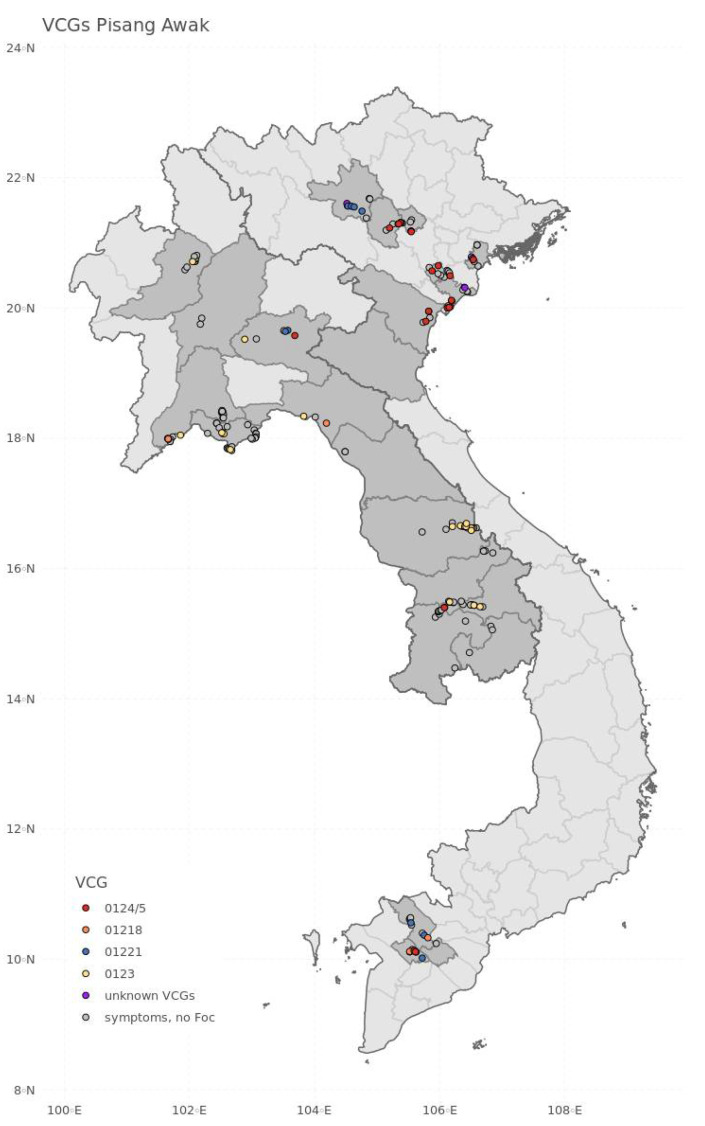
Observations of various VCGs associated with ‘Pisang Awak’. Grey points show ‘Pisang Awak’ fields which showed the traditional symptoms of wilt, but were not identified as fusarium. Red points show fields where VCG 0124/5 was positively identified; yellow points where VCG 0123 was identified; blue points where VCG 01221 was identified; orange points where VCG 01218 was identified.

‘Pisang Awak’ was infected by several VCGs of Foc R1/R2 (Figure 3, Table 3). In Laos, VCG 0123 was most common and was recorded in 20 fields. VCGs 0124, 01218 and 01221 were each isolated from ‘Pisang Awak’, each recorded in only two fields. In Vietnam, ‘Pisang Awak’ was infected with VCG 0124/5 in 17 fields, with VCG 01221 in seven fields, and with VCG 01218 in four fields. On two occasions, more than one Foc VCGs were identified in a single field. This included VCGs 0124 and 01218 in a ‘Pisang Awak’ small-holder farm in Can Tho, and VCGs 01213/16 and 0124 in a Cavendish/ ‘Pisang Awak’ small-holder farm in Phu Tho. Four isolates obtained from ‘Pisang Awak’ plants that amplified with Foc Lineage VII-specific primers did not pair with known VCG testers in this Lineage (Table 3). A single field with symptomatic ‘Pisang Mas’ plants was infected with VCG 0124, whereas the few symptomatic triploid BBB cooking bananas sampled were not infected by Foc. All Foc strains collected from Cavendish banana plants were identified as Foc TR4 (VCG 01213/16). Foc TR4 race was not isolated from ‘Pisang Awak’, ‘Pisang Mas’ or the triploid BBB cooking banana.

Fusarium wilt was found in all types of farms surveyed in Laos and Vietnam, including backyard gardens, small-holder farms and large-scale plantations. In both 2018 and 2020 surveys, Foc TR4 was only associated with large-scale Cavendish banana farms in Laos, whereas it was mainly recorded on small-scale Cavendish farms in Vietnam. Foc R1/R2 was collected from ‘Pisang Awak’ plants in Laos in 13 out of 65 backyard gardens, in eight of the 35 small-holder farms, and in five of 18 commercial plantations. In Vietnam, Foc R1/R2 was found in 18 of the 43 backyard gardens investigated, in nine of the 31 small-holder farms and in four of the six ‘Pisang Awak’ commercial plantations investigated.

The interviews conducted during the 2020 survey revealed that banana farmers were generally unaware of the threat posed by Fusarium wilt. Only four of the 120 farmers interviewed in Laos had heard of Fusarium wilt prior to the interview but did not know the cause of the disease. Three of these farmers could identify leaf yellowing and stem rot as symptoms. These farmers received the information from neighboring farmers. In Vietnam, only one of the 121 interviewed farmers knew of Fusarium wilt. Their knowledge of the disease was gained through the internet. This farmer was very well informed of Foc epidemiology. None of the farmers in Laos or Vietnam performed any pest or disease management and were unaware of biosecurity measures or quarantine regulations in their region. New planting material was mainly sourced as suckers; namely, 86.5% of interviewed farmers in Laos sourced new suckers from neighboring fields, and 10.1% from another village. In Vietnam, 94.2% of interviewed farmers sourced new suckers from neighboring fields. The remaining farmers obtained new material as tissue culture-derived plantlets.

## 4. Discussion

Foc TR4 was first detected in Luang Namtha and the Vientiane provinces of Laos in 2017 [32] and in Hanoi, Hung Yen and Lao Cai in Vietnam in 2014 and 2015 [33]. The current study has confirmed the presence of Foc TR4 in these provinces, but also the dissemination of Foc TR4 into additional regions in both countries. In Laos, a substantial number of Foc TR4-infested Cavendish fields have now further been identified in the northwestern provinces of Bokeo, Luang Prabang, Oudomxay and Xaignabouly. In Vietnam, most TR4-infested Cavendish fields were observed in the previously identified provinces of Hanoi, Hung Yen and Lao Cai, with additional scattered detections in the northern provinces of Lai Chai, Phu Tho, Hai Phong and Vinh Phuc and the southern provinces of Tay Ninh and Long An. It is believed that the expanding Cavendish cultivation by Chinese companies in the two countries with material sourced from potentially Foc TR4-infested plantations, without strict quarantine measures, could have led to pathogen introduction. Once introduced, local and regional trade of planting material, bunches and other plant parts, and the movement of farm equipment and laborers, could have resulted in its spread to other regions in the countries.

In Laos, Chinese private banana companies manage Cavendish plantations across the country. Between 2016 and 2017, a total of 117 Chinese companies established banana farms in Laos, covering 26,177 hectares across the country [46]. These farms were in northern (Bokeo, Luang Namtha, Oudomxay, Laung Prabang, Xaignabouly and Phongsaly), central (Vientiane, Vientiane Capital and Bolikhamxai) and southern Laos (Salavan, Champasak, Sekong and Attapeu) [47]. The number of companies has since dropped to 90 on 20,408 hectares [46], mainly due to the rise of Foc TR4 cases [47]. Bananas in affected northern regions were either replaced with other crops or production was moved to the center and south of Laos [47], potentially distributing Foc TR4 with them. Since no Foc TR4 was observed in the southern region of Laos, it can be assumed that the companies are increasingly focused on biosecurity measures to prevent the entry of the pathogen into the new production regions.

In Vietnam, Chinese private banana companies are mainly located in the North West. The occurrence of Foc TR4 in fields in the northern provinces can, however, not be directly linked to Chinese companies, as Foc TR4 was mainly detected on small-scale Cavendish farms. It is assumed that the pathogen reached these farms through local and regional movement of infected planting materials. In addition, the inoculum could also have spread widely in northern Vietnam through the Red River and its tributaries which all start in or run through infected Cavendish production zones in southern China. In China, the rapid spread of Foc TR4 was attributed to infected planting materials and the use of irrigation water [48]. Su et al. [49] and Dita et al. [9] also reported Foc spread through contaminated irrigation water or floods to be common. In Northern Vietnam, the Lao Cai province borders China, whereas the Phu Tho, Vinh Phuc, Ha Noi, Hai Phong and Hung Yen provinces are part of the Red River delta zone. The absence of Foc TR4 in the central and coastal provinces of Vietnam can possibly be explained by the limited cultivation of Cavendish bananas in these areas.

Thus far, in both Laos and Vietnam, Foc TR4 is limited to Cavendish cultivation settings, and none of the other investigated banana cultivars (‘Pisang Awak’, ‘Pisang Mas’ or the triploid BBB cooking banana) were infected with Foc TR4. This supports the hypothesis that currently Foc TR4 in the GMS is mainly spread through infected Cavendish planting material movements, and other Cavendish-based commercial and management activities, e.g., irrigation. ‘Pisang Awak’, a popular banana variety in Laos and Vietnam, and predominant in smallholder settings, was only infected by VCGs belonging to Foc R1/R2. The geographical distribution of VCG 0123 (R1) across Laos, and VCG 0124/5 (R1/2) throughout Vietnam, suggests that the movement of infected planting material and soil in the two countries is the most likely means of spread on smallholder settings. Grower’s surveys conducted in 2020 confirmed that new banana fields are primarily established with suckers from neighboring farms. The diversity of Foc VCGs associated with ‘Pisang Awak’ (VCGs 0123, 0124/5, 01218 and 01221) further suggests that Fusarium wilt is well established in the region. The same VCGs were previously reported in this region, with their occurrence closely associated with the banana variety being affected [50]. Without active prevention measures, it can be expected that Foc TR4 will spread more widely in the banana-based landscapes and start infecting other *Musa* cultivars grown in mixed cultivar arrangements, and subsequently through local trade.

Several *Fusarium* isolates collected from bananas in Laos and Vietnam could not be identified as known Foc VCGs and should be further investigated. Leaf yellowing, typical to Fusarium wilt, can be caused by biotic and abiotic stresses other than Fusarium wilt, such as waterlogging, nutrient deficiencies and bacterial diseases [51]. Non-pathogenic *F. oxysporum* strains are often isolated from diseased banana material [52], which may complicate the identification process, as it cannot be distinguished from Foc based on morphological characters or multi-gene phylogenetics. Other *Fusarium* spp., such as *F. proliferatum, F. solani* and *F. semitectum*, are also often associated with bananas [53,54]. It is, therefore, important to perform pathogenicity testing in addition to molecular identification and VCG testing when studying *Fusarium* spp. associated with banana.

Most of the farmers interviewed during the 2020 surveys were unaware of the cause of leaf yellowing symptoms in their banana fields, and none had any disease management practices in place. The most important practice for growers is to prevent the introduction of Foc into new areas by limiting the movement of infected material, farm equipment and/or laborers. Although governmental policy and support is critical to achieve this, effective implementation can only be achieved through improved communication and extension services. An additional level of complication for farmers can come from the fact that the symptoms of leaf yellowing are not solely associated with Fusarium wilt. During the surveys conducted in this study, Foc TR4 was isolated from only 29.2% of banana fields where samples were collected, which implies that a better understanding of causes of leaf yellowing in banana fields is required. To better manage Fusarium wilt in Laos and Vietnam, especially in Cavendish plantations biosecurity practices and extension services need to be improved. Where Foc TR4 has been introduced, susceptible Cavendish cultivars could potentially be replaced with partially resistant somaclones [22,55]. However, the use of these somaclones as a standalone innovation is not a guarantee of success. Complementary management practices, such as early detection and plant eradication, the use of biocontrol agents and soil health-oriented practices, amongst others, would still be needed to better cope with Fusarium wilt in fields established with these somaclones.

## Figures and Tables

**Table 1:** Overview of the 2018 survey. The number of districts, villages and fields are indicated for each province surveyed. The number of fields in which *Fusarium oxysporum* f. sp. *cubense* TR4 was positively identified are indicated. Foc TR4 was confirmed using a TR4-specific PCR primer [39].

Country	Macro-Region	Region	Province	Districts	Villages	Fields	Fields TR4 Confirmed
Vietnam	Northern Vietnam	North West	Lào Cai	3	4	10	8
			Lai Châu	2	3	7	1
			Sơn La	5	8	15	0
		Red River Delta	Hà Nội	4	5	16	8
			Hưng Yên	3	5	11	4
	Central Vietnam	North Central	* Nghệ An	3	10	22	0
		South Central	Quảng Ngãi	3	7	14	0
	Southern Vietnam	South East	Bình Phước	2	4	6	0
			Đồng Nai	3	4	10	0
			Tây Ninh	5	8	12	1
		Mekong River Delta	Cần Thơ	3	5	13	0
			Long An	4	8	11	1
	Total			40	71	147	23
Laos	Northern Laos	North West	Bokeo	2	6	14	10
			Luang Namtha	2	4	17	17
			Luang Prabang	3	6	20	6
			Oudomxay	3	5	10	6
			Phongsaly	3	4	10	0
			Xaignabouly	2	2	10	7
	Central Laos	Central	Vientiane Capital	3	5	10	2
			Vientiane	3	5	22	13
			Bolikhamxai	1	3	6	0
	Southern Laos	South East	Attapeu	2	3	17	0
			Salavan	2	2	11	0
			Sekong	1	1	8	0
	Total			27	46	155	61

* Provinces indicated in bold are surveyed in both the 2018 and 2020 surveys.

**Table 2:** Survey overview of the 2020 survey. The number of districts, villages and fields are indicated for each province surveyed. The number of fields in which *Fusarium oxysporum* f. sp. *cubense* (Foc) TR4 and Foc R1/R2 were positively identified are indicated. Foc TR4 and Foc R1/R2 were confirmed using a Foc TR4 or Foc R1/R2-specific PCR primers and Vegetative Compatibility Groups.

Country	Macro-Region	Region	Province	Districts	Villages	Fields	Fields TR4 Confirmed	Fields R1/R2 Confirmed
Vietnam	Northern Vietnam	North West	Yên Bái	3	7	10	0	5
		North East	Phú Thọ	4	7	11	3	5
		Red River Delta	Hà Nam	5	9	10	0	3
			Hải Phòng	2	7	10	1	2
			Nam Định	3	9	11	0	5
			Vĩnh Phúc	2	3	7	1	1
	Central Vietnam	North Central	* Nghệ An	1	1	1	0	0
			Thanh Hóa	3	6	7	0	3
	Southern Vietnam	Mekong River Delta	Cần Thơ	3	5	7	0	5
			Đồng Tháp	2	5	8	0	3
			Vĩnh Long	2	3	3	0	0
	Total			30	62	85	5	32
Laos	Northern Laos	North West	Luang Prabang	1	2	2	0	0
			Oudomxay	2	8	11	1	1
			Xiangkhouang	2	6	6	0	5
	Central Laos	Central	Vientiane Capital	6	22	28	1	3
			Vientiane	3	12	15	0	2
			Bolikhamxai	2	3	3	0	2
			Khammouane	1	2	2	0	0
			Savannakhet	3	16	19	0	9
	Southern Laos	South East	Attapeu	2	3	4	0	0
			Champasak	1	6	10	0	1
			Salavan	2	7	10	0	1
			Sekong	2	9	10	0	2
	Total			27	96	120	2	26

* Provinces indicated in bold are surveyed in both the 2018 and 2020 surveys.

**Table 3:** Vegetative compatibility group (VCG) identification of *Fusarium oxysporum* f. sp. *cubense* during the 2020 survey. VCG and race identification of samples isolated from specific cultivars. The number of fields in which these VCG cultivar associations are found are indicated per province.

Country	Region	Province	Banana Cultivar	Genome Group	Foc Race *	VCG	Fields
Vietnam	North West	Yên Bái	‘Pisang Awak’	ABB	R1	01221	4
		Yên Bái	‘Pisang Awak’	ABB	R1/R2	Unknown VCG	1
	North East	Phú Thọ	Cavendish	AAA	TR4	01213/16	3
		Phú Thọ	‘Pisang Awak’	ABB	R1	01218	1
		Phú Thọ	‘Pisang Awak’	ABB	R1/R2	0124/5	3
		Phú Thọ	‘Pisang Awak’	ABB	R1/R2	0124	1
	River Delta	Hà Nam	‘Pisang Awak’	ABB	R1/R2	0124/5	3
		Hải Phòng	Cavendish	AAA	TR4	01213/16	1
		Hải Phòng	‘Pisang Awak’	ABB	R1/R2	0124/5	1
		Hải Phòng	‘Pisang Awak’	ABB	R1/R2	Unknown VCG	1
		Nam Định	‘Pisang Awak’	ABB	R1	Unknown VCG	1
		Nam Định	‘Pisang Awak’	ABB	R1/R2	0124/5	2
		Nam Định	‘Pisang Awak’	ABB	R1/R2	0124	2
		Vĩnh Phúc	Cavendish	AAA	TR4	01213/16	1
		Vĩnh Phúc	‘Pisang Awak’	ABB	R1/R2	0124	1
	North Central	Thanh Hóa	‘Pisang Awak’	ABB	R1/R2	0124/5	1
		Thanh Hóa	‘Pisang Awak’	ABB	R1/R2	0125	1
		Thanh Hóa	‘Pisang Mas’	AA	R1/R2	0124	1
	Mekong River Delta	Cần Thơ	‘Pisang Awak’	ABB	R1	01218	2
		Cần Thơ	‘Pisang Awak’	ABB	R1	01221	1
		Cần Thơ	‘Pisang Awak’	ABB	R1/R2	0124	2
		Cần Thơ	‘Pisang Awak’	ABB	R1/R2	Unknown VCG	1
		Đồng Tháp	‘Pisang Awak’	ABB	R1	01218	1
		Đồng Tháp	‘Pisang Awak’	ABB	R1	01221	2
Laos	North West	Oudomxay	Cavendish	AAA	TR4	01213/16	1
		Oudomxay	‘Pisang Awak’	ABB	R1	0123	1
		Xiangkhouang	‘Pisang Awak’	ABB	R1	01221	2
		Xiangkhouang	‘Pisang Awak’	ABB	R1	0123	2
		Xiangkhouang	‘Pisang Awak’	ABB	R1/R2	0124	1
	Central	Vientiane Capital	Cavendish	AAA	TR4	01213/16	1
		Vientiane Capital	‘Pisang Awak’	ABB	R1	0123	3
		Vientiane	‘Pisang Awak’	ABB	R1	01218	1
		Vientiane	‘Pisang Awak’	ABB	R1	0123	1
		Bolikhamxai	‘Pisang Awak’	ABB	R1	01218	1
		Bolikhamxai	‘Pisang Awak’	ABB	R1	0123	1
		Savannakhet	‘Pisang Awak’	ABB	R1	0123	9
	South East	Champasak	‘Pisang Awak’	ABB	R1/R2	0124	1
		Salavan	‘Pisang Awak’	ABB	R1	0123	1
		Sekong	‘Pisang Awak’	ABB	R1	0123	2

* R1, R2 and TR4 are different races.

## Data Availability

The raw data supporting the conclusions of this manuscript will be made available by the authors, without undue reservation, to any qualified researcher.

## Supplementary Materials

The following are available online at https://www.mdpi.com/article/10.3390/jof8010046/s1, Figure S1: Diagram illustrating characterization strategy of *Fusarium oxysporum* f. sp. *cubense* strains collected from Vietnam and Laos during the 2020 surveys, Figure S2: Amplification products of duplex PCRs using genomic DNA from pure-cultures with *Fusarium oxysporum* f. sp. *cubense* (Foc)-like appearance. Duplex PCRs for Foc cultures were performed using the elongation factor−1α (EF-1/EF-2) primer set as internal control in combination with the TR4-specific primer FocTR4-F/FocTR4-R. Specific DNA bands for Foc TR4 (463 bp) and elongation factor 1α (648 bp) are indicated on the right. Lanes N, P and 3–24, respectively, denote negative control (water), positive control (Foc TR4 gDNA) and gDNA of assessed plant samples. Foc cultures for detecting the presence or absence of TR4 were isolated from banana plant samples obtained from plants with Fusarium wilt characteristic symptoms on farms in Laos, Figure S3: Vegetative compatibilty between representative isolations from Laos and Vietnam and *Fusarium oxysporum* f. sp. *cubense* universal VCG testers.
