# Supplementary material for: Geographical Distribution and Genetic Diversity of the Banana Fusarium Wilt Fungus in Laos and Vietnam
Source: J Fungi (Basel). 2022 Jan 2;8(1):46. doi: 10.3390/jof8010046 (PMC8781582; doi:10.3390/jof8010046)
Supplement: Supplementary file 1 [file jof-08-00046-s001.zip › jof-1465898-supplementary.pdf]

Journal of *Fungi*

MDPI

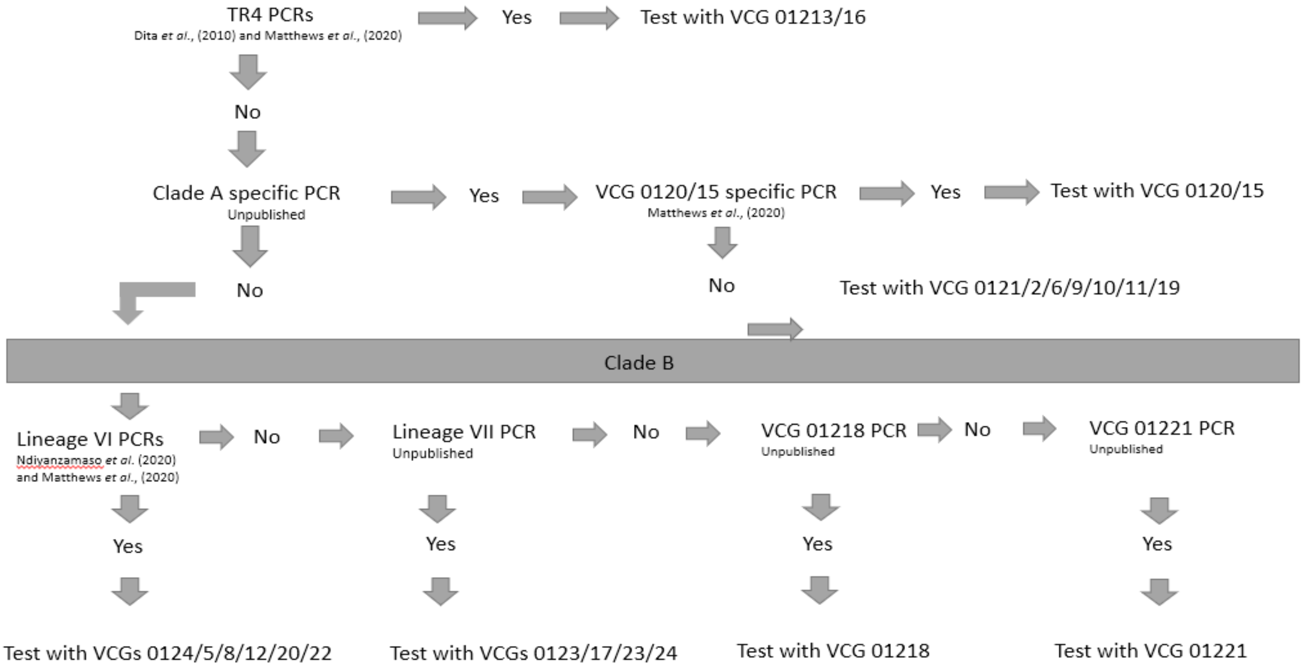

Supplementary Figure S1. Diagram illustrating characterization strategy of *Fusarium oxysporum* f. sp. *cubense* strains collected from Vietnam and Laos during the 2020 surveys.

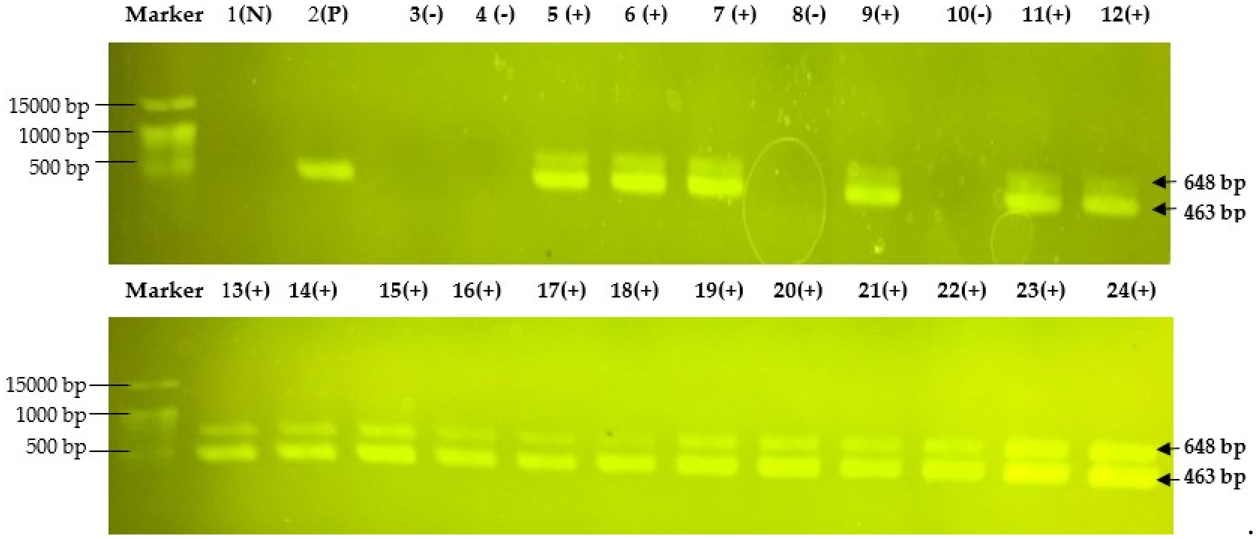

Supplementary Figure S2: Amplification products of duplex PCRs using genomic DNA from pure-cultures with *Fusarium oxysporum* f. sp. *cubense* (Foc) like appearance. Duplex PCRs for Foc cultures were performed using the elongation factor-1α (EF-1/EF-2) primer set as internal control in combination with the TR4-specific primer FocTR4-F/FocTR4-R. Specific DNA bands for Foc TR4 (463 bp) and elongation factor 1α (648 bp) are indicated on the right. Lanes N, P and 3-24, respectively, denote negative control (water), positive control (Foc TR4 gDNA) and gDNA of assessed plant samples. Foc cultures for detecting presence or absence of TR4 were isolated from banana plants samples obtained from plants with Fusarium wilt characteristic symptoms on farms in Laos.

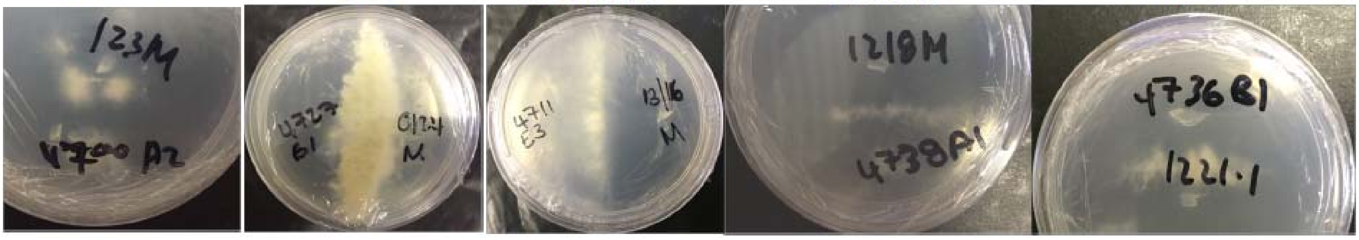

Supplementary Figure S3: Vegetative compatibilty between representative isolations from Laos and Vietnam and *Fusarium oxysporum* f. sp. *cubense* universal VCG testers.
